# Supplementary material for: TANGO2 is an acyl-CoA binding protein
Source: J Cell Biol. 2025 Feb 27;224(5):e202410001. doi: 10.1083/jcb.202410001 (PMC11867700; doi:10.1083/jcb.202410001)
Supplement: Table S1 — shows the identification (A to I) and measurement analysis of each cavitation’s area, volume, and deepness in angstrom (Å) by KVFinder predictor. [file jcb_202410001_tables1.docx]

| KVFinder Predictor | | | | |
| --- | --- | --- | --- | --- |
| Cavity Identification | Area  (Å^2^) | Volume  (Å^3^) | Average Deep (Å) | Maximum Deep (Å) |
| A | 78.66 | 73.22 | 0.99 | 3.7 |
| B | 144.87 | 126.36 | 1.35 | 4.37 |
| C | 41.34 | 31.1 | 0.76 | 2.4 |
| D | 63.11 | 68.47 | 0.97 | 3 |
| E | 15.08 | 6.7 | 0.18 | 0.85 |
| F | 625.99 | 851.26 | 3.87 | 14.76 |
| G | 13.46 | 7.78 | 0.3 | 0.85 |
| H | 64.53 | 55.73 | 1.31 | 2.81 |
| I | 19.35 | 13.61 | 0.39 | 1.34 |
