# Supplementary material for: TANGO2 is an acyl-CoA binding protein
Source: J Cell Biol. 2025 Feb 27;224(5):e202410001. doi: 10.1083/jcb.202410001 (PMC11867700; doi:10.1083/jcb.202410001)
Supplement: SourceData F1 — is the source file for Fig. 1. [file jcb_202410001_sourcedataf1.pdf]

FIGURE 1

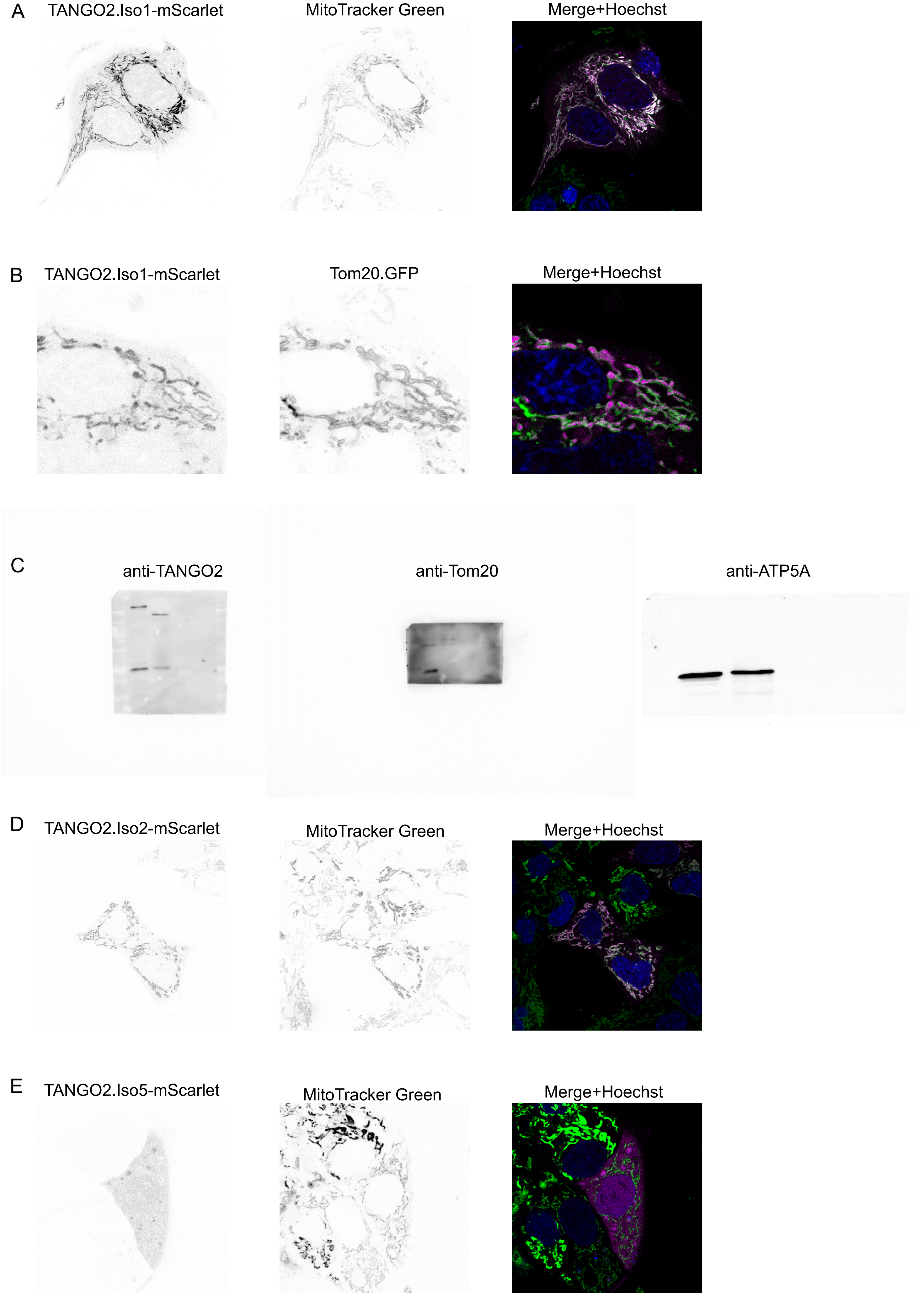
A
TANGO2.Iso1-mScarlet
MitoTracker Green
Merge+Hoechst
B
TANGO2.Iso1-mScarlet
Tom20.GFP
Merge+Hoechst
C
anti-TANGO2
anti-Tom20
anti-ATP5A
D
TANGO2.Iso2-mScarlet
MitoTracker Green
Merge+Hoechst
E
TANGO2.Iso5-mScarlet
MitoTracker Green
Merge+Hoechst
