# Supplementary figures and images for: TANGO2 is an acyl-CoA binding protein
Source: J Cell Biol. 2025 Feb 27;224(5):e202410001. doi: 10.1083/jcb.202410001 (PMC11867700; doi:10.1083/jcb.202410001)

FIGURE 2

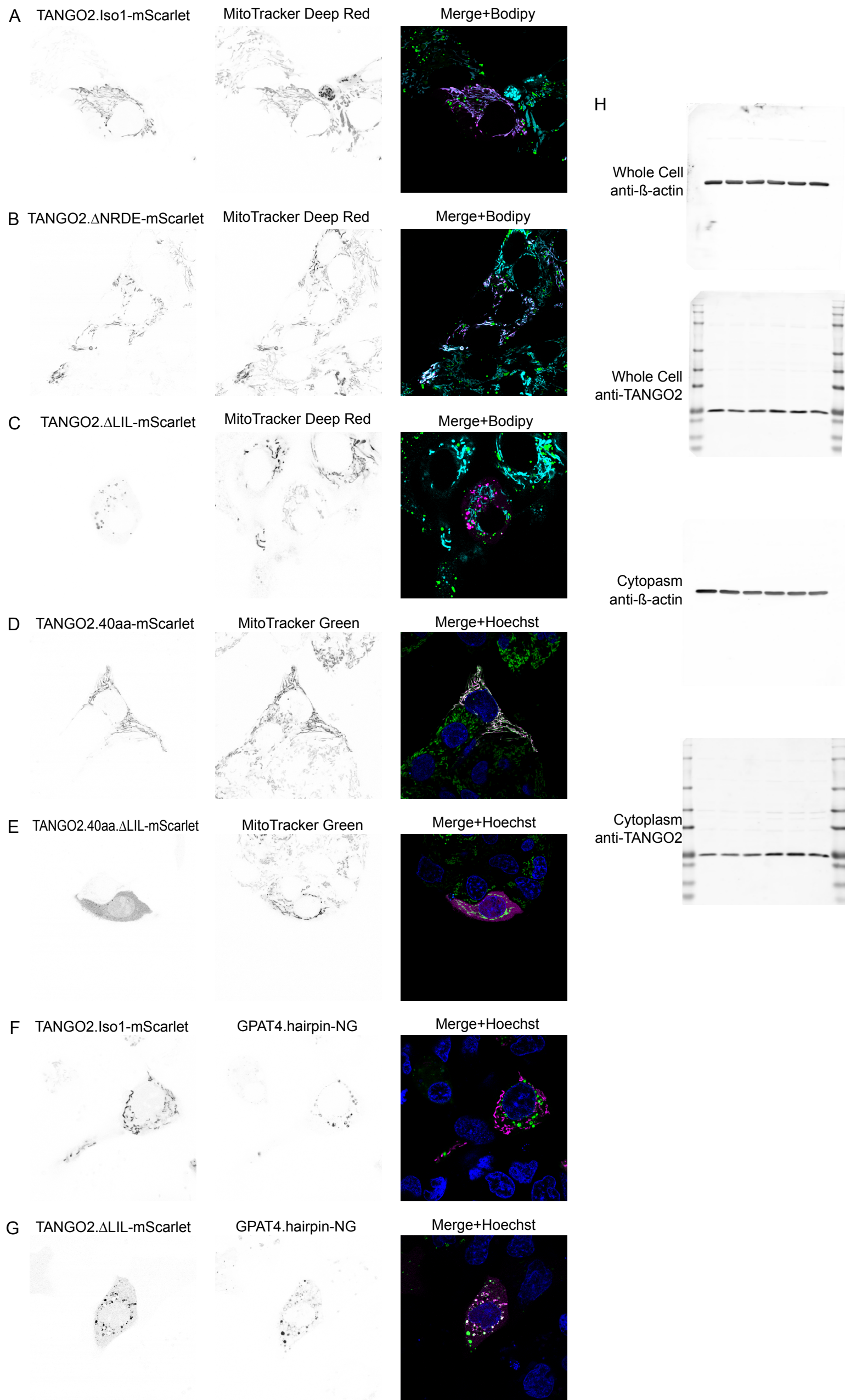

Supplement: SourceData F2 — is the source file for Fig. 2. [file jcb_202410001_sourcedataf2.pdf]

FIGURE 4

B

anti-RFP

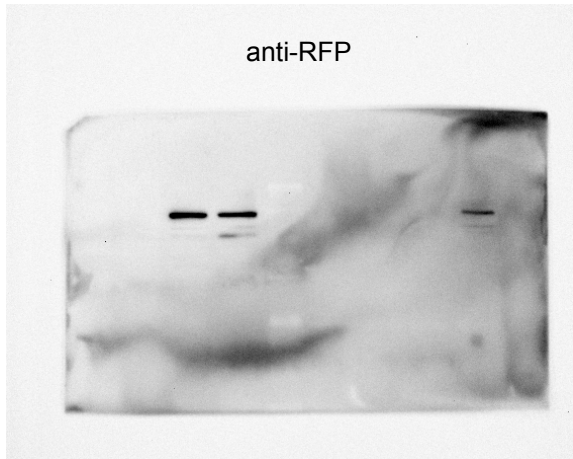

anti-Flag

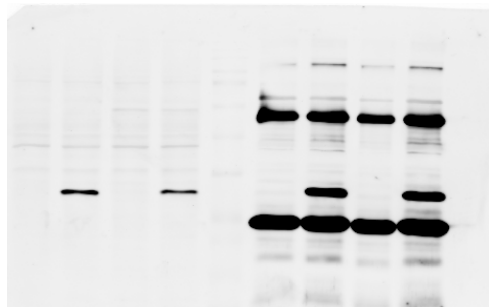

anti- $\beta$ -actin

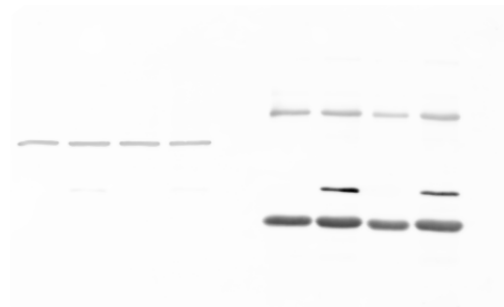

Supplement: SourceData F4 — is the source file for Fig. 4. [file jcb_202410001_sourcedataf4.pdf]

# SUPPLEMENTARY FIGURE 1

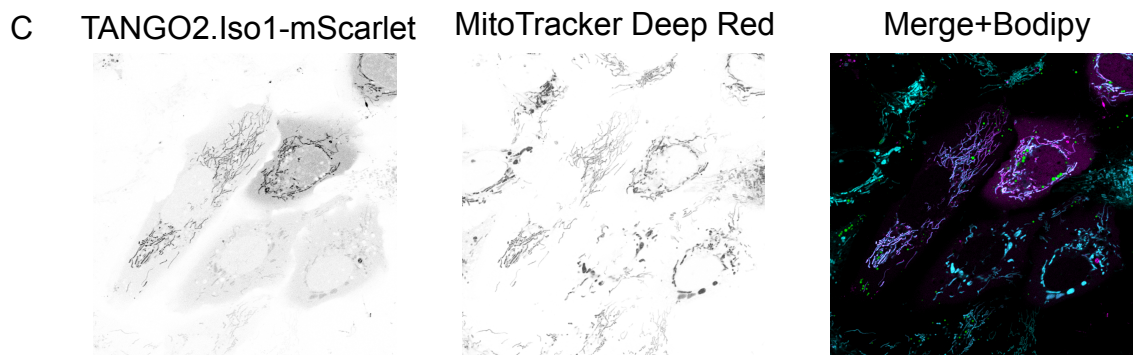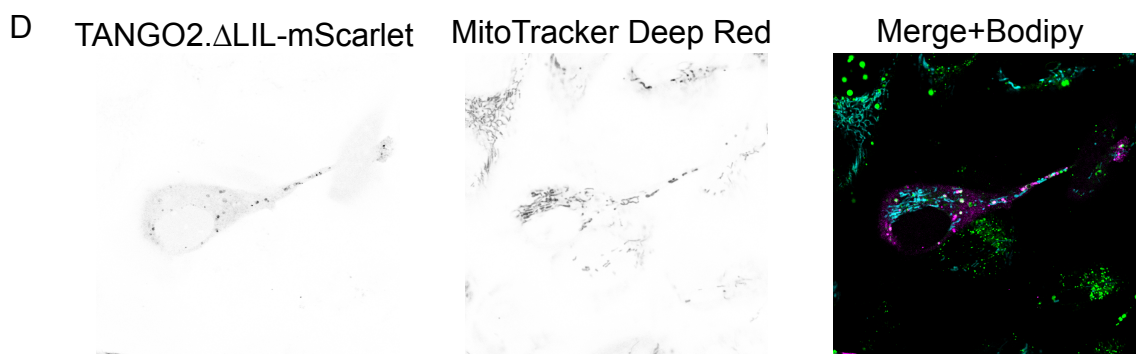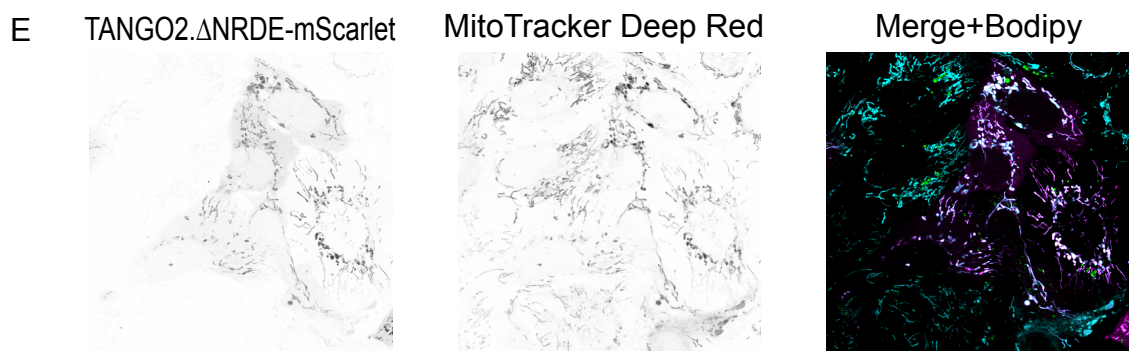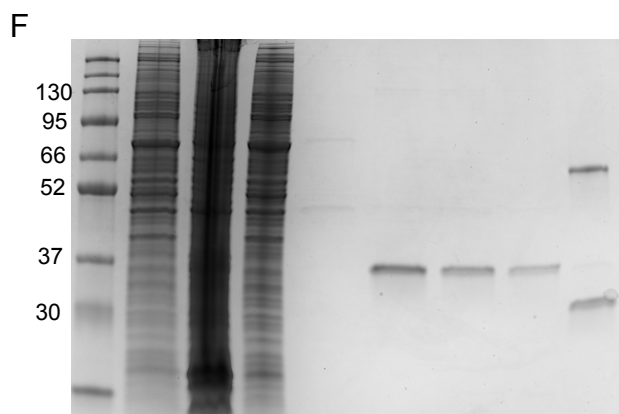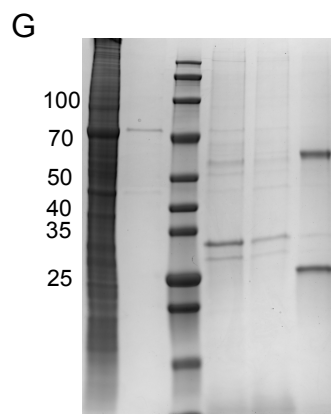

Supplement: SourceData FS1 — is the source file for Fig. S1. [file jcb_202410001_sourcedatafs1.pdf]
